# Book Reviews

**Published:** 1916-05

**Authors:** 


### Book Reviews.

Social Travesties and What They Cost. By D. T. Atkinson, M. D. The Vail-Ballou Co., New York.

This book by Dr. Atkinson, of Dallas, is receiving favorable comment everywhere. The following article from the *Dallas News* of April 3d expresses so concisely our own opinion of the book that we take the liberty of reprinting it in full:

"The value of this treatment of the social evil from the physician's standpoint lies not so much in the original matter which the author has contributed to it, though that is negligible neither in quantity or quality, but in the fact that he has brought together all the salient features of medical research from authoritative sources, in compact, easily get-atable form. So much of importance that has been produced on the subject is hidden from lay readers in purely professional treatises that to embrace the gist of it in a book of this scope and in a manner that appeals to the general reader, is a distinct service to society. This service the author has ably performed. The subject is particularly vital to parents and to young men and women and boys and girls who have reached the age of discretion, and the manner in which the facts have been presented makes the book suitable for general use in extending the social purity propaganda. Dr. Atkinson is a Dallas physician and specialist, and the fact that many of his observations have been drawn from his own practice forcibly brings home the dangers from which no community or class is immune."

Doctor—"You have nervous dyspepsia, same as Brown had. His was caused by worrying over his butcher's bill. I directed him to stop worrying."

Stranger—"Yes, and now he's cured, and I've got it. I'm his butcher."—*Boston Transcript.*

